# Author's reply

**Published:** 2010

**Authors:** Pushpa Sharma, Brandi Benford, Zhao-Zhang Li

**Affiliations:** Department of Anesthesiology, Uniformed Services University of the Health Sciences, Bethesda MD 20814, USA psharma@usuhs.mil

Sir,

I am writing in response to the questions raised by one of our readers.[1] First, I would like to thank him for taking an interest in our publication.[2] Since the acceptance of this paper, we have generated some interesting data on how our dipstick test compares with conventional spectrophotometric tests for pyruvate dehydrogenase content and enzyme activity assessment.

I agree with the reader that complete evaluation of the dipstick test in the diagnosis of traumatic brain injury has to be done, and studies using various animal models with compromised mitochondrial functions are in progress in our laboratory. As stated in the original publication,[2] this dipstick test is highly sensitive when compared to the spectrophotometric test because only 25 μg protein is needed; the spectrophotometric method requires at least 50-75 μg protein, which can be a problem when only a small quantity of sample is available. Like any other diagnostic test, the dipstick test results may also vary with the type of tissue analyzed, method of tissue collection, handling and storage processes, as well as the underlying pathophysiological conditions. We are also examining the influence of these factors on pyruvate dehydrogenase activity in our current studies. Also, to avoid the pre–analytical error, we have standardized the cell lysis protocol and equal volumes of samples are being used to avoid variation in dipping techniques.

As indicated in the original article, the dipstick cost only $10 per test, can be done quickly, and is easy to do. In comparison, the conventional spectrophotometric test costs at least $50 per test and needs a trained scientist or technician to perform the pyruvate dehydrogenase measurement.

In conclusion, this dipstick test is in its early stages of development and we are working to validate the diagnostic capability of this potentially useful tool.
